# Addendum: RAF/MEK/extracellular signal–related kinase pathway suppresses dendritic cell migration and traps dendritic cells in Langerhans cell histiocytosis lesions

**DOI:** 10.1084/jem.2016188107092026a

**Published:** 2026-07-24

**Authors:** Brandon Hogstad, Marie-Luise Berres, Rikhia Chakraborty, Jun Tang, Camille Bigenwald, Madhavika Serasinghe, Karen Phaik Har Lim, Howard Lin, Tsz-Kwong Man, Romain Remark, Samantha Baxter, Veronika Kana, Stefan Jordan, Zoi Karoulia, Wing-hong Kwan, Marylene Leboeuf, Elisa Brandt, Helene Salmon, Kenneth McClain, Poulikos Poulikakos, Jerry Chipuk, Willem J.M. Mulder, Carl E. Allen, Miriam Merad

Vol. 215, No. 1 | https://doi.org/10.1084/jem.20161881 | December 20, 2017

The authors wish to clarify that duplication of the “BRAFV600E + MEKi” plot in Fig. 3 B and the “MEKi” plot in Fig. 3 G was intentional. These panels represent the same experimental dataset, which was deliberately reused to facilitate comparison across different treatment conditions. Specifically, in panel B, the plot shows *BRAF*V600E bone marrow–derived dendritic cells treated with a MEK inhibitor. In panel G, the identical plot was intentionally included as the reference MEK inhibitor condition (right panel) to allow direct comparison with the ABT-treated condition (middle panel) using the same experimental dataset. This was indicated in the figure legend; however, the authors recognize that the wording could have been clearer. The authors apologize for any confusion and appreciate the opportunity to clarify this point.

**Figure 3.**
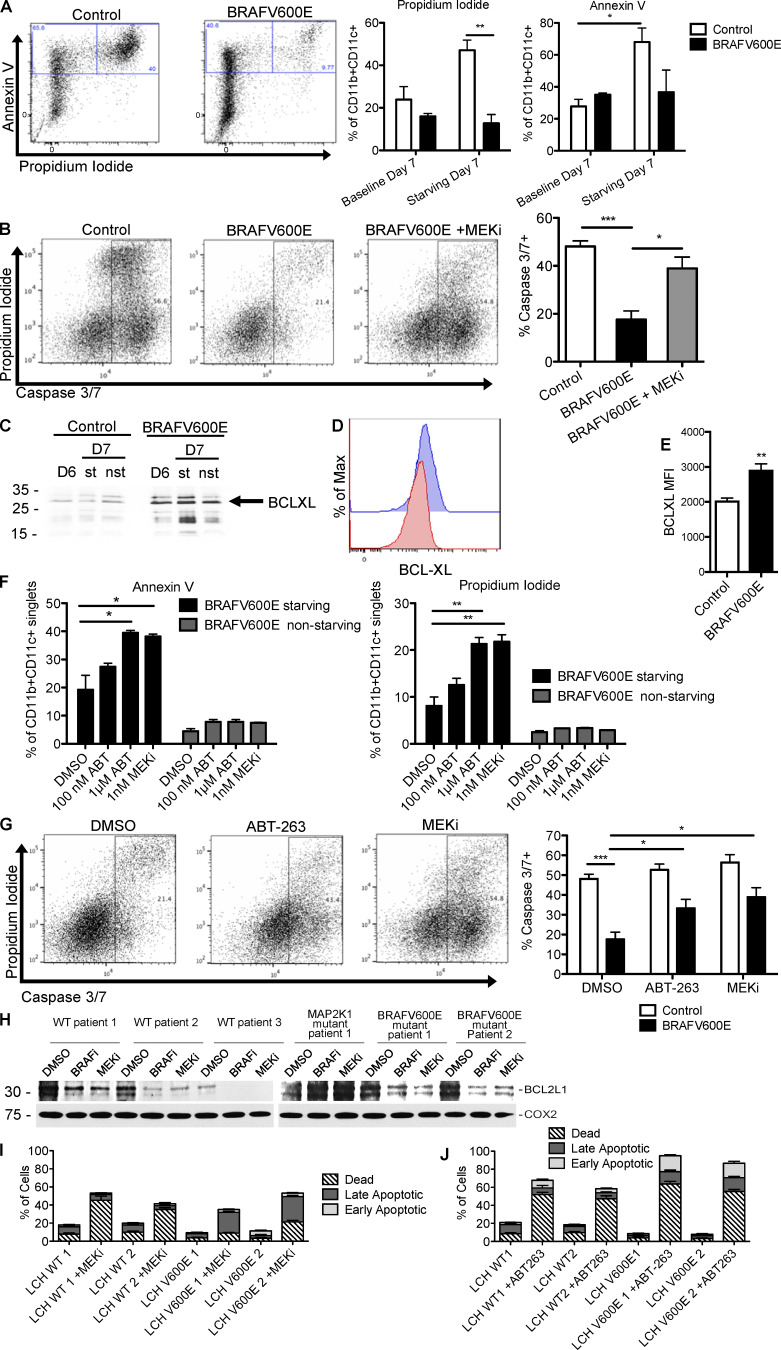
**BCL-XL up-regulation via RAF/MEK/ERK signaling contributes to suppressed apoptosis in DCs. (A)** Apoptosis measured in control and *BRAF*V600E BMDCs by Annexin V/propidium iodide (PI) staining. On D6 of culture, GM-CSF cytokine was removed from the BMDC culture, and BMDC viability was measured on D7 by flow cytometry. Dot plots show representative frequency of apoptotic Annexin^+^PI^+^ CD11c^+^CD11b^+^ BMDCs from two experiments. Bar graphs show the mean of triplicate samples representative of two experiments ± SEM (*n* = 3–5; control vs. *BRAF*V600E starved PI positivity: **, P = 0.0055; unpaired *t* test; baseline vs. starved control Annexin V positivity: *, P = 0.0419; unpaired *t* test). **(B)** Caspase 3/7 activation measured in control and *BRAF*V600E BMDCs starved of the GM-CSF growth factor overnight (***, P = 0.0008; unpaired *t* test), ±1 nM GSK1120212 (*, P = 0.0161; unpaired *t* test). Representative samples are shown in FACS plots. Bar graphs show the mean of three biological replicates representative of two experiments ± SEM. **(C)** BCL-XL expression was measured by western blot in *BRAF*V600E^CD11c^ BMDCs starved (st) or not starved (nst) of the GM-CSF growth factor in overnight culture and harvested on D6 or D7 of culture. Representative data from two independent experiments are shown. **(D and E)** BCL-XL protein levels in control and *BRAF*V600E BMDCs as measured by flow cytometry. Representative data from at least two experiments with three biological replicates are shown. The bar graph in E represents quantification of triplicate conditions within one experiment ± SEM (**, P = 0.0089; unpaired *t* test). **(F)** Percentage of apoptotic BMDCs among control or *BRAF*V600E BMDCs cultured overnight with 100 nM BCL2-family inhibitor ABT-263, 1 µM ABT-263 (Annexin V: *, P = 0.0211; unpaired *t* test; PI: **, P = 0.0032; unpaired *t* test), or 1 nM GSK1120212 MEKi (Annexin V: *, P = 0.0268; unpaired *t* test; PI: **, P = 0.0030; unpaired *t* test). BMDCs were starved or nonstarved of the GM-CSF growth factor during overnight drug treatment and analyzed for apoptosis using Annexin V/PI staining by flow cytometry. Bar graphs show the mean of three biological replicates ± SEM, representative of two independent experiments. **(G)** Caspase 3/7 activation measuring *BRAF*V600E BMDCs treated with a control vehicle (***, P = 0.0004; unpaired *t* test) or with 1 nM GSK1120212 (*, P = 0.0118; unpaired *t* test), as shown in B, or in the presence of 1 µM ABT-263 (*, P = 0.0330; unpaired *t* test) overnight. Bar graphs show the mean results of triplicate conditions from two independent experiments ± SEM. **(H)** Western blot showing BCL2L1 protein levels in human LCH lesions cultured without serum overnight, then treated with BRAF or MEKi’s for 2 h. (C and H) Molecular mass is indicated in kilodaltons. **(I and J)** Viability of human LCH lesions cultured overnight without serum, then treated for 2 h with 1 nM GSK1120212 MEKi (I) or 1 µM ABT-263 BCL2-family inhibitor (J). Three patient samples are shown in each treatment group. Data represent means shown ± SEM. D6, day 6; D7, day 7.

